# For whom is a health-promoting intervention effective? Predictive factors for performing activities of daily living independently

**DOI:** 10.1186/s12877-016-0345-8

**Published:** 2016-10-06

**Authors:** Synneve Dahlin-Ivanoff, Kajsa Eklund, Katarina Wilhelmson, Lina Behm, Greta Häggblom-Kronlöf, Lena Zidén, Sten Landahl, Susanne Gustafsson

**Affiliations:** 1 Department of Health and Rehabilitation, The Sahlgrenska Academy at the University of Gothenburg, Institute of Neuroscience and Physiology, Box 455, SE 405 30 Gothenburg, Sweden; 2 Institute of Neuroscience and Physiology, University of Gothenburg Centre for Ageing and Health (AgeCap), SE 405 30 Gothenburg, Sweden; 3 Department of Health Sciences, Faculty of Medicine, Lund University, SE 221 00 Lund, Sweden; 4 Department of Geriatrics, The Sahlgrenska University Hospital, Blå stråket 5, SE 413 45 Göteborg, Sweden

**Keywords:** Aged, 80 and over, Frail elderly, Health promotion, Activities of daily living (ADL), Logistic regression, Ageing

## Abstract

**Background:**

Health-promoting interventions tailored to support older persons to remain in their homes, so-called “ageing in place” is important for supporting or improving their health. The health-promoting programme “Elderly Persons in the Risk Zone,” (EPRZ) was set up for this purpose and has shown positive results for maintaining independence in activities of daily living for older persons 80 years and above at 1- and 2 year follow-ups. The aim of this study was to explore factors for maintaining independence in the EPRZ health-promoting programme.

**Methods:**

Total of 459 participants in the original trial was included in the analysis; 345 in the programme arm and 114 in the control arm. Thirteen variables, including demographic, health, and programme-specific indicators, were chosen as predictors for independence of activities of daily living. Logistic regression was performed separately for participants in the health promotion programme and in the control arm.

**Results:**

In the programme arm, being younger, living alone and self-rated lack of tiredness in performing mobility activities predicted a positive effect of independence in activities of daily living at 1-year follow-up (odds ratio [OR] 1.18, 1.73, 3.02) and 2-year, (OR 1.13, 2.01, 2.02). In the control arm, being less frail was the only predictor at 1-year follow up (OR 1.6 1.09, 2.4); no variables predicted the outcome at the 2-year follow-up.

**Conclusions:**

Older persons living alone — as a risk of ill health — should be especially recognized and offered an opportunity to participate in health-promoting programmes such as “Elderly Persons in the Risk Zone”. Further, screening for subjective frailty could form an advantageous guiding principle to target the right population when deciding to whom health-promoting intervention should be offered.

**Trial registration:**

The original clinical trial was registered at ClinicalTrials.gov. Identifier: NCT00877058, April 6, 2009.

**Electronic supplementary material:**

The online version of this article (doi:10.1186/s12877-016-0345-8) contains supplementary material, which is available to authorized users.

## Background

The current trend in Western societies facing a growing proportion of older persons is to support them to remain in their homes for as long as possible, so-called “ageing in place” [1]. This justifies the focus in this paper, were attention is directed to explore factors that predict independence in everyday life for community-dwelling older persons when attending health-promotion intervention.

Older persons constitute a group whose reserve of physical strength is decreasing, and whose participation in activities of daily life (ADL) will deteriorate with increasing frailty [2, 3]. However, these declines notwithstanding, engagement in ADL continues and has a beneficial effect on older persons’ sense of self [4]. This beneficial effect was confirmed by Haak et al. [5], who also found that independence in ADL among older persons is strongly and positively associated with ageing in place and that independence is highly valued and reinforces feelings of pride and satisfaction. Independence in ADL is also considered an important indicator of health [5–7]. Dependence, in contrast, is correlated with an increased risk of dying [7] and poor quality of life [6] among older persons. The transition from independence in ADL to any form of dependency is a crucial threshold for older persons. Therefore, it is essential to enable older persons to continue performing daily activities independently in their own homes even as frailty progresses. To support or improve health, it is important to develop effective preventive interventions tailored to older persons at risk of becoming frail. The “Elderly Persons in the Risk Zone” (EPRZ) health-promoting programme [8] has shown positive results for maintaining independence in daily activities. To understand the effectiveness of health-promotion for the target group, it is important to explore which factors predict a successful outcome in terms of independence in ADL in order to offer the intervention to those who benefit most from it.

A review of health-promotion initiatives targeting older persons highlighted the lack of evidence for superiority of one format of intervention over another [9]. The reported value of one particular format, preventive home visits targeting older people in ordinary housing, has varied among different studies [10–12]. Some studies have documented impact on mortality, nursing home admissions, falls, functional decline, and ADL [10, 13–16]; however, a recent systematic review showed no clear evidence that preventive home visits were effective in preventing loss of physical and psychosocial function, falls, nursing home admissions, or death [17]. Another format is group intervention, but few studies have evaluated the impact of this intervention. Nevertheless, these studies have shown positive results [18–20].

The EPRZ health-promoting programme aimed to prevent limitations in ADL and to support ageing in place for community-dwelling older persons at risk of becoming frail. The EPRZ study was a randomized controlled trial (RCT) with follow-ups for up to 2 years, and was designed to evaluate the impact of two formats of health-promotion interventions; a preventive home visit (PHV) and senior group meetings (SM). The results from this study showed that both formats had positive effects; the PHV reduced dependency in ADL at the 1-year follow-up [21], and participants in the SM experienced both short- and long-term reductions (3 months, 1- and 2 years) [21, 22]. Qualitative investigations of the EPRZ health-promoting programme add to these results by showing a generally positive reaction to participation in both intervention formats. The PHV brought feelings of security and gave participants an incentive to action [23], and, the SM was perceived as a key to action, e.g. keep being active in daily life [24].

In conclusion, both health-promotion intervention formats in the EPRZ programme showed positive effects in slowing dependence in ADL after one year, and the SM intervention extended this advantage for up to 2 years [21]. The dissimilar design of the two interventions might be an explanatory factor for their impact. However, it is possible that factors beyond the format of health-promotion intervention could aid in prediction of a successful outcome in terms of independence in ADL for older persons (aged ≥ 80 years) at risk of becoming frail. Other factors, such as demographics (e.g. living condition and age) or health-related factors, such as frailty, risk of depression and life satisfaction, might play crucial roles for the proven favorable effects of health-promotion for the target group. Therefore, the aim of this study was to explore factors for maintaining independence in the EPRZ health-promoting programme.

## Methods

### Research design and study population

This study involved secondary analysis of longitudinal data from the health-promoting RCT EPRZ [8]. The study was conducted in two urban districts in Gothenburg, Sweden, in which people over 80 years of age account for 8 % and 7 % of the population, compared with 5 % for Gothenburg as a whole and for Sweden as a whole. The two districts are situated outside the city centre but within the city limits, and contain a mix of single-family houses and apartment buildings. The general educational levels and income levels of residents were slightly higher, and the sickness rates somewhat lower, than in the general population of Gothenburg. The EPRZ study was a randomized, single-blind, three-armed trial (*n* = 459), with follow-ups for up to 2 years. The sample was intended to be representative of community-dwelling older persons, 80 years or older, at risk of becoming frail. Older persons living in ordinary housing, who were cognitively intact, and who did not require help from another person in ADL were included. A power calculation for the EPRZ study has been conducted [8]. The study was approved by the regional Ethical Board in Gothenburg (ref. no: 650–07).

In the EPRZ study, eligible participants were drawn from official registers of all persons over 80 years of age in the two urban districts in Gothenburg. Equal numbers from the two districts were listed in random order. The persons were included in the sample consecutively using the simple random sampling chart until the intended sample size was reached. For the 546 persons who consented to participate, a total of 459 persons were included: 174 in the PHV, 171 in the SM, and 114 in the control group. For more information, see the study protocol [8]. Main focus is on the two formats of health-promotion interventions (PHV or SM) (*n* = 345) due to their success in maintaining independence. The control group is included to check for similarity of predictors.

The main aim of the EPRZ health-promoting programme was to delay the progression of frailty, ADL dependence and morbidity in older persons at risk of becoming frail in order to support ageing in place. The programme was performed in collaboration among an occupational therapist, a physical therapist, a registered nurse, and a social worker, all of whom were employed in the two urban districts. The basic foundation, consistent with a person-centred [25] and empowerment [26] approach, was that older persons themselves were seen as experts, and were encouraged to make autonomous decisions and, as far as possible, to control their own lives. The PHV included a single home visit that provided information about services available for older citizens in the urban district, e.g., physical training groups, accessibility to assistive devices and housing modifications, and the identification of environmental fall risks in the home. The PHV was guided by a protocol, which included an opportunity to elaborate on certain elements according to each person’s experience and needs. The home visit lasted between 90 min and 2 hours. The SM comprised four senior group meetings followed by one follow-up home visit after 2–3 weeks. The SM provided an arena for the exchange of knowledge and information, discussion about the ageing process, possible health consequences, and strategies for solving the various problems that may arise in the home environment. Predetermined themes, outlined in a booklet written in a popular style by researchers in the field and especially designed for the intervention, formed a basis for the meetings [27]. The EPRZ health-promoting programme was implemented essentially as planned. All participants assigned to the PHV received the intervention. Ninety-seven percent of the participants (165 of 171) in the SM attended all four meetings.

Data at baseline and 1- and 2-year follow-ups, were collected in face-to-face structured interviews conducted by blinded research assistants during home visits [8]. All interviews included a comprehensive assessment (Additional file 1). All interviewers were well trained in interviewing, assessing, and observing, according to the guidelines for the different outcome measurements. To ensure as much standardization of the assessments as possible, study protocol meetings were held regularly throughout the study.

### Measurements

#### Dependent variable

Independence of another person in ADLs was assessed according to a cumulative scale of well-defined personal and instrumental activities, the ADL staircase [28]. Nine of the 10 original activities: cleaning, shopping, transportation, cooking, bathing, dressing, going to the toilet, transferring, and feeding were used (0–9). The variable was dichotomised into “Independence” (in all included activities) and “Dependency” (in any one of the included activities) in line with the importance of the transformative threshold from independency to any form of dependence. Data concerning the dependent variable were assessed by face-to-face interview and observation according to guidelines at 1- and 2 year follow-ups.

#### Explanatory variables

Thirteen explanatory variables (eight health-related, four demographic, and one programmatic) were selected by the responsible multidisciplinary research group on the basis of the importance of their influence on the outcome measure, which is a recommended practice [29]. The selected health-related variables were: frailty, morbidity, self-rated health, risk of depression, tiredness in mobility activities, perceived security in ADL, life satisfaction, and loneliness. Selected demographic variables were: age (continuous), sex (male/female), living conditions (living alone/cohabiting) and education (lower/higher, with higher education defined as university or college). Data on all explanatory variables were collected at baseline, before participant’s exposure to the health-promoting programme. For details, see the EPRZ study protocol [8]. Format of health-promotion (PHV or SM) was also included as a programmatic variable because of the differences between the interventions.

*Frailty* was measured by means of eight core frailty indicators: (1) weakness, operationalised as grip strength and measured by North Coast dynamometer below 13 kg for females and below 21 kg for males for the dominant hand, and below 10 kg for women and 18 kg for males for the non-dominant hand; (2) fatigue, defined as an affirmative answer to the question: “Have you suffered any general fatigue/tiredness over the last 3 months?”; (3) weight loss, defined as an affirmative answer to the question: “Have you suffered any weight loss over the last 3 months?”; (4) low physical activity, defined as 1–2 walks/week or less; (5) poor balance, defined as 47 or lower on Berg’s balance scale; (6) low gait speed, defined as walking 4 m or less in 6.8 s; (7) visual impairment, defined as a visual acuity of < 0.5 in both eyes using the KM chart; and (8) cognition, defined as < 25 points on the Mini Mental State Examination. For more details, see the EPRZ study protocol [8]. Report of three or more frailty indicators was, according to recommendations by Fried [30], used as a cut-off for the dichotomization of “Frailty”/”No frailty” in this study.

*The Mob-T Scale* [31] was used to measure tiredness associated with performance of the following mobility activities: (1) walking indoors, (2) getting outdoors, (3) transferring to or from a bed or chair, (4) walking outdoors in nice weather and (5) in bad weather, and (6) managing stairs, with three response options: perform without tiredness, perform with tiredness, or too tired to perform the activity. The variable was dichotomised into “No tiredness” (performing all daily activities without tiredness) and “Tiredness” (performing any of the daily activities with tiredness or too tired to perform the activity).

*Morbidity/disability* was measured with the Cumulative Illness Rating Scale for Geriatrics (CIRS-G) [32], a quantitative rating instrument of the chronic medical illness burden that has been modified for geriatric assessment. CIRS-G contains 14 categories: heart; vascular; hematopoietic; respiratory; eyes, ears, nose, throat, and larynx; upper gastrointestinal; lower gastrointestinal; liver; renal; genitourinary; musculoskeletal; neurological; endocrine; and psychiatric illness. The rating reflects both degree of morbidity and disability. The rating scale ranges from 0–4, with (0) indicating no problem; (1) a current mild problem or past significant problem; (2) a moderate disability or morbidity requiring “first line” therapy; (3) a severe or constant significant disability or “uncontrollable” chronic problems; and (4) end-organ failure or severe organ function impairment requiring extremely critical or immediate treatment. In this study, consistent with other research [33], ratings of 2 or greater in each category were recoded as 2, and the variable was dichotomized for each participant as “≤ 2 ratings of 2” and “≥ 3 ratings of 2”.

*Self-rated health* was measured by the single question [34]: “In general, would you say your health is: excellent, very good, good, fair or poor? In this study, “Good health” was defined as excellent, very good, and good, and “Bad health” as fair or poor.

*Risk of depression* was measured by the Geriatric Depression Scale (GDS) [35]. A Swedish modified version was used, including 20 items with “yes” or “no” responses. One point is assigned for each”yes” response. Scores from 0–5 are interpreted as “No risk of depression,” and scores above 5 as “Risk of depression” [36].

*Perceived security in ADL,* from the Instrumental and Personal Activities of Daily Living (I- and P-ADL) [28], was used to measure insecurity in bathing, dressing, going to the toilet, transferring, feeding, cleaning, shopping, cooking and transport. The response alternatives were (1) secure, (2) fairly secure, (3) insecure, and (4) very insecure. To dichotomise this variable, the highest level of perceived security (response alternative 1) in all included activities was used as a cut-off.

*Satisfaction with life as a whole* was measured by the one overall item in the Fugel–Meyer-Life Satisfaction Assessment (LiSat-11) [37]. The participants estimated their satisfaction on a six-grade scale, which was dichotomised into “Satisfied” (for responses of rather satisfied, very satisfied, and satisfied) or “Dissatisfied” (for responses of rather dissatisfied, dissatisfied, or very dissatisfied).

*Loneliness* was measured with the question: “Do you feel lonely?”, and dichotomized as “Yes” (for responses of rarely, sometimes, or often) or “No” (for the response of never).

### Statistical analysis

All 13 explanatory variables were entered into binary logistic regression with backward elimination of variables with the smallest contribution to the model to find the set of independent variables with the highest explanatory power for ADL. The analysis was conducted per protocol according to recommended assumptions regarding regression [38]. To find out if the explanatory variables also were valid for the control group; the same analysis was performed separately for the control group (*n* = 114). Statistical analyses were performed using PASW Statistics, version 22.0 (IBM SPSS Inc., Chicago, IL, 2009).

## Results

All participants in the sample used for this study were independent in ADL at baseline. After 1 year, 197 participants (57 %) in the programme arm were still independent, and the number was 130 (38 %) after two years. The dropout rate was 12 % at 1-year follow-up and 21 % at 2-year follow-up. The main reason for discontinuation, other than death (*n* = 12), was non-interest or lack of time (*n* = 19). There was no significant difference between participants and dropouts in demographic data at baseline. Characteristics of the participants at baseline are presented in Table 1.

**Table 1:** Characteristics of participants in the EPRZ study and control

Characteristics	Program arm (*n* = 345)	Control arm (*n* = 114)
Median age (range)	85 (80–94)	86 (80–97)
Female, *n* (%)	224 (65)	69 (61)
Living alone, *n* (%)	202 (59)	55 (48)
Academic education^a^, *n* (%)	72 (21)	24 (22)

^a^Tertiary education (University or College)

The backward logistic regression showed that being younger (odds ratio [OR] 1.16), living alone (OR 1.73), and performing mobility activities without tiredness (OR 3.02) were baseline predictors of a successful outcome of the interventions in terms of independence in ADL at the 1-year follow-up (Table 2).

**Table 2:** Predictors of independence in ADL, at one- and two year follow-ups in the program arm, in the EPRZ study

	One year follow-up			Two year follow-up		
Explanatory variables	Prob. Chi-Sq	OR	95 % CI	Prob. Chi-Sq	OR	95 % CI
Demographic						
Younger age	**< 0.00**	**1.16**	**1.08 to 1.25**	**< 0.00**	**1.2**	**1.04 to 1.2**
Living alone	**0.03**	**1.73**	**1.07 to 2.79**	**< 0.00**	**2.22**	**1.37 to 3.61**
Higher education	0.73	0.9	0.5 to 1.62	0.76	0.91	0.51 to 1.64
Gender	0.44	1.2	0.71 to 2.2	0.92	1.03	0.59 to 1.81
Programmatic						
Programme format	0.70	0.9	0.57 to 1.45	0.36	1.24	0.78 to 1.97
Health-related						
Lack of tiredness in mobility activities	**< 0.00**	**3.02**	**1.86 to 4.90**	**0.006**	**2.02**	**1.22 to 3.33**
Frailty	0.09	0.82	0.65 to 1.03	0.99	1.00	0.57 to 1.76
Morbidity	0.82	1.02	0.86 to 1.21	0.20	0.89	0.75 to 1.06
Self-rated health	0.88	0.95	0.46 to 1.94	0.67	1.17	0.56 to 2.47
Risk of depression	0.38	1.45	0.63 to 3.34	0.36	0.67	0.28 to 1.59
Perceived security in ADL	0.4	0.77	0.43 to 1.40	0.50	1.23	0.67 to 2.51
Life satisfaction	0.06	2.16	0.96 to 4.88	0.7	1.18	0.52 to 2.70
Loneliness	0.69	1.13	0.62 to 2.08	0.73	1.11	0.61 to 2.0

The significant predictors are marked in bold

The regression also showed that being younger (OR 1.13), living alone (OR 2.01), and performing mobility activities without tiredness (OR 2.02) at baseline were predictors of successful outcome at the 1-year follow-up (Table 2). None of the identified predictors that explained a successful outcome in maintaining independence among participants exposed to the EPRZ health-promoting programme explained a successful outcome in the control group. Instead, in the control group, being less frail (OR 1.6, CI 1–1 to 2.4) was found to be a predictor at 1 year but there was no predictor found at 2 years (Table 3). For the control group, 51 persons (45 %) remained independent in ADL after 1 year, and 40 (35 %) after 2 years.

**Table 3:** Prob. Chi-Sq for independence in ADL, at one- and two year follow-ups in the control group

Explanatory variables	One year follow-up	Two year follow-up
Demographic		
Younger age	0.12	0.86
Living alone	0.92	0.21
Higher education	0.68	0.42
Gender	0.32	0.83
Health-related		
Lack of tiredness in mobility activities	0.48	0.48
Frailty	**0.02**	0.65
Morbidity	0.39	0.64
Self-rated health	0.73	0.15
Risk of depression	0.3	0.89
Perceived security in ADL	0.93	0.054
Loneliness	0.09	0.17

The significant predictors are marked in bold

## Discussion

The final model showed, at both 1- and 2-year follow-ups, that being younger and living alone were explanatory variables for a positive outcome in ADL for those attending the health promotion programme. Additionally, lower self-ratings of tiredness in performing mobility activities at both follow-ups predicted higher levels of independence in ADL. None of these were found to be valid for the control arm. The only predictor found for maintaining independence in the control arm was at 1-year follow up; being less frail.

The fact that living alone was a significant predictor of outcome at both the 1- and 2-year follow-ups was somewhat unexpected. Research has shown that living with a spouse in old age is a positive health factor [39], and that older persons living alone are more likely to report negative health experiences, for example difficulties in instrumental and personal ADL [40]. A more intuitive explanation would be that living with a spouse would emerge as an explanatory variable, but this was not the case in our analysis. Instead, the health-promoting programme appears to have had a greater benefit for those who lived alone, a known risk group for ill health, and a group that in this study was found to have a two times higher chance of independence in ADL two years after exposure to the interventions than those living with a spouse. Both health-promoting intervention formats in the EPRZ programme seems to have given participants a push in the right direction (an incentive for healthier lifestyle choices) [23, 24], and may thus have offered support, at the right time, for this risk group. An alternative explanation might be that older persons living on their own, must cope with everyday activities on their own and therefore may be more motivated for behaviour change than those who can rely on a spouse. The EPRZ programme might have provided them with tools and strategies for a better and more effective mastering of ADL on their own. Although there may be other explanations for the finding that living alone was a predictor, we find the explanation that a known risk group for ill health was reached and had the most benefit from the health-promoting programme, the most likely.

Age—specifically, being younger—was also a significant predictor at the 1- and 2-year follow-ups. Being younger, in this context, can be defined as being in the lower end of the age continuum of participants in the EPRZ study (median 85.6, range 80–94). This finding raises the question of timing: at what age should health-promotion for community-dwelling older persons be offered for optimal effects? An overly simplified answer to this question based on the results would be to recommend a lowering of the target age for such interventions to, for example, 75 years or older. Moreover, research has shown that those who benefit most from a health-promotion intervention are those who are not too frail [41, 42]. Therefore, although frailty did not emerge as a predictor in our analysis, and considering the relationship between age and frailty [30], variations in the degree of frailty among community-dwelling older persons [43] need to be taken into account when deciding whom to target with an intervention. Additionally, according to qualitative studies [23, 24], an older person’s own opinion of the right timing of a health-promotion intervention is important for the intervention to be perceived as valuable by the individual. Consequently, a more person-centred approach [25], in which an older person’s own perception of the appropriate starting point for health-promotion is taken into consideration in partnership (e.g. between the older person and a health care-professional). An assessment of older persons (tentatively 75–80 years or older) including screening of frailty and ADL status in combination with age, and taking the individual’s own opinion of timing into account, might constitute useful guiding principles for targeting a health-promotion intervention. Such screening and its importance for preventive actions are also supported by others [44]. To conclude, simply lowering the target age for health-promotion initiatives addressing community-dwelling older persons will probably not guarantee a better outcome if these other factors of frailty, ADL status, and the individual’s own opinion of timing are not taken into consideration.

The last exploratory variable to be discussed is tiredness during mobility activities. Participants who reported performing mobility activities without tiredness had three respective two times greater chance of maintaining independence in ADL at the 1- and 2-year follow-ups. A possible explanation could be that tiredness in mobility activities, as measured by the Mob-T scale [31], captures an early, self-reported dimension of frailty. As such, the emergence of this variable as a predictor in our study fits very well into the above discussion of intervention being more beneficial for those in early stages of frailty. This also supports the use of a screening tool as a preface for proposing a health-promotion intervention to an older person. Such a screening tool should include an early indicator and subjective measure of frailty, such as the Mob-T scale. In addition, the experience of fatigue when performing mobility activities may indicate that an older person has entered a “sedentary” phase. It is known that reduced physical activity increases risk of morbidity, such as cardiovascular disease [45], Osteoporosis [46], Diabetes [47] and Depression [48]. It is also hard to break a vicious cycle of fatigue–reduced activity–impaired health, a fact that may adversely affect health-promotion initiatives for older persons experiencing tiredness during mobility activities. Another possibility is that those who feel tired during such activities have begun to develop diseases that are not yet fully visible or diagnosed.

The fact that the format of the intervention (PHV or SM) did not predict outcomes must be addressed. This finding is in line with a review of health-promotion interventions targeting older persons, which found no evidence that one format of intervention was better than another [9]. The review, together with results from the previously discussed qualitative studies [23, 24], indicates that the older person’s choice of format, based on personal needs and preferences, is what ought to dictate the type of health-promotion intervention.

Finally, since none of the identified explanatory factors could explain maintaining independence in ADL within the control group at any follow-up, it strengthens our findings. These three explanatory factors seem to be particularly successful factors for those who have a favorable intervention outcome. Within the control group, those being less frail from the start had a higher chance of maintaining independence. This is not surprising, as frailty increases with age and the strong relationship between frailty and dependence in ADL. But, the control group is small, possibly affecting statistical power. Thus, this interpretation should be held with some caution.

To summarise, persons 80 years and older are a vulnerable group whose reserve of strength is failing, and just a small change can result in the transition threshold of becoming dependent in ADL. The health-promoting programme EPRZ, which offers information about ageing and provides tools and strategies to strengthen participants’ capabilities to age in place, was set up to meet this need. This study showed that being younger, living alone, and performing mobility activities without tiredness predicted a positive outcome of independence in ADL after participating in the health-promoting programme. The identification of predictive factors can guide the planning of future implementations of EPRZ, and aid the development and research of similar health-promoting initiatives. To capture the target group that benefits most from an intervention is of value for the society in terms of used resources but most importantly for the individual in terms of supporting ageing in place.

Regarding the method of this study, there are some issues that need to be discussed. First, study dropout rates tend to rise with participants increasing age, and this may have affected the generalisability of the study’s results [38]. For the sample in this study, the dropout rates were 12 % and 21 % at the 1- and 2-year follow-ups, respectively. These numbers should be considered acceptable and should not affect generalisation, because attrition rates of up to 30 % in RCTs including older persons are considered tolerable according to an acclaimed organisation assessing study quality [49]. Even so, the dropout rates in combination with the by time lessening residual group of independent participants constituted a general motivation for dichotomising variables in this study in order to achieve robustness of the result. Clearly, the dichotomisation entails a risk for oversimplification of actual circumstances, but, this must be weighted by the fact that the power calculation was done for the EPRZ RCT, and not for the secondary analyses in this study.

Also, there is the issue of reasons for attrition. In this study sample, as well as in the whole EPRZ RCT, the two main reasons were death and unwillingness to continue because of a lack of interest or time. The intention was that EPRZ should comprise a representative sample of community-dwelling persons 80 years or older who are at risk of becoming frail. But, it is questionable whether a possible unfulfilled intention could impact on the generalisability of the results. The two main causes for dropout can be seen as extreme ends of a spectrum, resulting in remaining participants representing a narrow “middle population” that is neither too ill (close to death) nor among the most vigorous (lack of time = healthy and fully occupied). The attrition can thus be classified as non-random [50], and consequently, generalisability of results is uncertain. And, although the study’s longitudinal design contributed to some attrition, such designs also have several positive effects. In our secondary analysis, it allowed for observation of the temporal sequence between the baseline and the outcome after 1 and 2 years. The pattern that the predictors were invariant over time could not have been detected if a cross-sectional design had been applied.

There are also specific strengths and weaknesses associated with the selection of variables for the analysis. A strength is that during the selection process, the variables were discussed and debriefed by the responsible multidisciplinary research group, which is a recommended practice [29]. These discussions were, in turn, guided by the variables’ described importance of influence on ADL as an outcome measure [51, 52]. It is also important to consider a possible impact of a potential conceptual overlap between two variables (frailty and morbidity) in relation to ADL. Such overlap could pose a risk of multicollinearity effects. However, this idea is opposed by research showing that frailty, morbidity, and dependence in ADL are interrelated but different concepts [53]. Another potential conceptual overlap with possible impact on results is between insecurity in ADL and dependence in ADL. But, as in the former case, studies show that these two are mutually distinct, each measuring separate constructs [54]. In discussing the variables, it is also important to highlight that the measures were derived from well-validated instruments for the dependent as well as predictor variables.

The last methodological aspect in need of review is the importance of considering the width of confidence intervals (CIs) when interpreting results [38]. In general, the CI values in this study were rather narrow. A narrow CI could indicate that the sample represented a homogenous group [55], an argument that can be supported by the earlier discussion of possible impact of a non-random attrition. However, a narrow CI can be seen as producing a higher precision of the analysis and less uncertainty in the point estimate, and thereby a result with higher quality [38].

Finally, the appraisal of this study’s possible methodological shortcomings, as well as its outcome, should be made in the light of two important conditions. First, according to our knowledge, this study is the only one of its kind. We have not found any other study exploring factors that may predict a successful outcome in terms of independence in ADL for older persons after participating in health-promotion. Second, this study includes a follow-up of up to 2 years. This enhances explanatory power and makes the contribution to the field of knowledge within health-promotion for older community-dwelling persons more refined than a traditional cross-sectional study.

## Conclusions

Older persons living alone — as a risk of ill health — should be especially recognized and offered an opportunity to participate in health-promoting programmes such as “Elderly Persons in the Risk Zone”. Further, screening for subjective frailty could form an advantageous guiding principle to target the right population when deciding to whom health-promoting intervention should be offered.

## Additional file

Additional file 1: Summary of the Elderly Persons in the Risk Zone (EPRZ) assessment form. (DOCX 42 kb)
