# Supplementary material for: For whom is a health-promoting intervention effective? Predictive factors for performing activities of daily living independently
Source: BMC Geriatr. 2016 Oct 6;16:171. doi: 10.1186/s12877-016-0345-8 (PMC5052718; doi:10.1186/s12877-016-0345-8)
Supplement: Additional file 1: — Summary of the Elderly Persons in the Risk Zone (EPRZ) assessment form. (DOCX 42 kb) [file 12877_2016_345_MOESM1_ESM.docx]

**Summary of the Elderly Persons in the Risk Zone (EPRZ) assessment form**

Each group of questions/statements and instruments/tests have different answering alternatives (not shown). Also, some questions/statements have follow-up questions (not shown). Table 1 presents a condensed summary with the study time line for follow-ups.

**Demographic questions**

- Year of birth, sex, marital status?
- How and with whom do you live?
- What education do you have?
- What was your profession before retirement?

**Environmental/accessibility questions**

- Can you get in and out of your home without having to climb stairs?
- Do you experience difficulties to get in and out of your home due to obstacles in the environment?
- Do you experience difficulties in the area of your residence due to obstacles in the environment?
- Is your home adapted to your needs?

**Instruments/tests**

- The Mobility-Tiredness scale
- Hand strength
- The Berg balance scale
- Time to walk 4 meters
- The CIRS-G
- The Geriatric Depression Scale
- The MMSE
- The KM visual acuity chart
- How is your hearing?
- The FES-I
- Have you fallen sometimes in the last three months?

**Lifestyle questions**

- Do you smoke and/or use snus/snuff?
- How often do you drink alcohol?
- Specify how often you walk or engage in other similar physical activity?
- How long does the walk/physical activity usually last?

**Quality of life and health**

- The Göteborg quality of life instrument
- The EQ-5D
- Self-rated health
- The Fugl-Meyer life satisfaction scale

**Medications**

**Daily activities, participation and assistive technology**

- The ADL-staircase
- Technical aids?
- The Activity checklist

**Meal habits**

- Do you usually have breakfast, lunch and/or dinner?
- Do you usually have snacks in between meals?
- How often do you have vegetables for your meals?

**Social network and support**

- Do you have children?
- How often do you have contact with your children and/or grandchildren?
- Do you have someone to trust and confide in?
- Who do you turn to first to ask for help if you fall ill and bedridden?
- Sometimes you need help and support from someone. Do you have a relative, friend
  or another person you can turn to if you needed practical help?
- Do you feel alone?
- Do you feel more or less alone now than 10 years ago?
- Are you giving help to/assist someone in daily life activities?

**Health care consumption**

*Table 1.* Outcome measurements and follow-ups in EPRZ.

| **Outcomes** | **Measurement** | **TO** | **T1** | **T2** | **T3** |
| --- | --- | --- | --- | --- | --- |
|  |  |  | **3 month** | **1 year** | **2 year** |
| Fatigue | Questionnaire/tiredness scale | X | X | X | X |
| Grip strength | North Coast dynamometer | X |  | X | X |
| Endurance/physical activity | Questionnaire/physical and | X | X | X | X |
|  | activity scale | X | X | X | X |
| Balance | The Berg Balance Scale | X | X | X | X |
| Gait speed | Gait speed four-meter walking test | X | X | X | X |
| Weight loss | The Göteborg Quality of Life Instrument | X | X | X | X |
| Cognition | Mini Mental State Exam (MMSE) | X |  | X | X |
| Visual impairment | KM visual acuity chart | X |  | X | X |
| Self-rated health | SF 36 (a single question) | X | X | X | X |
| Illness | CIRS-G | X |  | X | X |
| Symptoms | The Göteborg Quality of Life Instrument | X | X | X | X |
| Depression | GDS 20 | X | X | X | X |
| Activities of daily living | The ADL staircase | X | X | X | X |
| Health-related quality of life | EQ5D | X | X | X | X |
| Life satisfaction | Fugl-Meyer - LiSat | X | X | X | X |
| Assistive technology and accessibility | Questionnaire | X | X | X | X |
| Participation/Leisure activities | Questionnaire | X | X | X | X |
| Social support | Questionnaire | X | X | X | X |
| Social network | Questionnaire | X |  | X | X |
| Falls | Questionnaire | X | X | X | X |
| Fear of falling | FES-I | X | X | X | X |
| Health care Consumption | Register data |  |  |  |  |
